# The History and Practice of Vaccination

**Published:** 1818-04

**Authors:** 


### PART II.

### COMPREHENSIVE ANALYTICAL REVIEW OF MEDICAL LITERATURE.

"Tros, tyriusve, nobis nullo discrimine agetur."

*The History and Practice of Vaccination.* By JAMES MOORE, Director of the National Vaccine Establishment, Surgeon of the 2d Regiment of Life Guards, and Member of the Royal College of Surgeons London. pp. 300. 8vo. London, 1817.

*Account of an Epidemic Small Pox, which occurred in Cupar in Fife, in the Spring of* 1817; *and the Degree of protecting Influence which Vaccination afforded; accompanied with Practical Inferences and Observations.* By HENRY DEWAR, M. D. F. R. S. E. and Fellow of the Royal College of Physicians, Edinburgh. pp. 38. 8vo. 1817.

AT a time like the present, when the hue and cry of prejudice is revived, in several populous districts, against the immortal discovery of the immortal Jenner, and when the columns of our public prints are filled with libels against vaccination, emanating from persons, who, though they cannot reason, and cannot pretend to any knowledge of physic, physiology, or therapeutics, to qualify them for such a delicate task, nevertheless discuss the question of the anti-variolous power of the vaccine lymph with the utmost impetuosity, not to say indecorum of language, and decide against it with all the plenitude of confidence inseparable from arrogant ignorance, it gives us more than ordinary pleasure to introduce these two Treatises to the acquaintance of our readers. They are the production of men of well known talents, inured, moreover, to habits of investigation as well as of ratiocination, and qualified by their knowledge, both special and collateral, to execute the task undertaken; and though they may fall short of silencing, far less of convincing, the

> "————————— Pharmacopolæ,

> Mendici, mimæ, balatrones, *hoc genus omne;*"

we mean, the reptile tribe of practice hunters, whether fluttering or creeping; the needy pamphleteers; wonder-mongers; and speech makers, each after his kind; who set themselves in opposition to vaccination either from lack of wit or from lack of money, and who, Heaven knows! are too far gone for argument; yet on all that is venerable and respectable in our community for rank, intellect, or influence, we anticipate that they will produce a most solid and indelible impression in favour of the efficacy of this most brilliant discovery! a discovery, which, in the eyes of posterity, will give greater eclat to this, our age, than the late tremendous conflicts of contending armies; the wondrous rise or fall of dynasties; or the upraising or upsetting of thrones.

Mr. Moore's is indeed an admirable work; judiciously planned, and ably executed; and will be perused by readers both in and out of the profession, with equal gratification; equal delight. Seldom, of a truth, does it fall to our lot, in these our analytical labours, to meet a book which we have been able to go through with so much unmingled pleasure, or rather gaiety. With much good sense and valuable practical information, there are conjoined much playful satire, which, nevertheless, scorches pretty severely; (a thing not to be regretted, considering *where* it lights), and some unexpected flashes of sly humour, which every now and then surprised us into a smile. Indeed, we are convinced, that a very respectable remnant of his father's *mantle* has descended in hereditary succession to our author; and it is paying the latter no common compliment, when we tell him, that we had not gone far into his volume, ere we recognized some traits of the writer of "Medical Sketches;"* and that we did not finish it, without being more than once reminded of that happiness of expression, and facility of style, that unobtrusive humour, poignant without coarseness, and chaste without tameness; that shrewdness of observation which was refined and ennobled by the spirit of philosophy till it became no longer *mere* shrewdness; and that unaffected taste for the polish and the graces of society, combined with a pure relish for the charities and the simplicities of human life, which constituted Dr. John Moore one of the most pleasing of writers, and one of the most amiable of men.

Real, therefore, as is our regard for our author, candour obliges us to say, that we are not sure but our feelings in

* By John Moore, M.D. author of Zeluco, &c. &c.

his favour are somewhat heightened by our own latent associations, and by those crowding recollections which are indissolubly connected with the name he has the honour to bear. Notwithstanding, we are equally free to confess our belief, that the native proper strength of the sapling will sufficiently support it without any aid from, or reference to, the parent oak ; and that, on the present occasion, the decision of impartial criticism will be, that Mr. James Moore has produced a book at once sensible, useful, and amusing ; so much so, indeed, that while it may be taken up as light reading, from its airy and jocular tone, — the frequent appearance of deep reflection and manly argumentation, will prevent the easy familiarity of the writer from arousing any thing like contempt in the Reader. For our own parts, we are no great advocates for the intermixture of Fun and Physic; yet, when it is discreetly done, as in the present case, it becomes so *piquante*, that

> " *Not* to laugh were want of goodness or of grace,"

and would argue in us more than even critical sourness and solemnity.

We must now hasten to lay before our Readers the more prominent information contained in this interesting volume: The notice of Dr. Dewar's Tract will come in more appropriately towards the close of the article.

The 1st. Chapter, entitled the "Discovery," contains some interesting biographical notices of Dr. Jenner, with an account of the tone of his mind, his early addiction to natural history, and his friendship with Sir Joseph Banks and Mr. John Hunter: by the latter, indeed, he was solicited to become a partner in business ; but well was it for the world, that accidental and family reasons induced him to decline this flattering offer, as it would, if accepted, have lost him those imperishable laurels which he afterwards gathered in the shades of Gloucestershire.

Having settled as a surgeon at Berkley, he commenced an inquiry about the cow-pox, which the popular belief of the county gave out as being an occasional preventative of the small-pox. After incredible difficulties, which would have proved, with ordinary minds, a barrier to all further investigation, he at last acquired a discriminating knowledge of the peculiar genuine vaccine disease, as observed on the teats of cows, or the fingers of the milk maids accidentally infected with it; and from one of the vesicles in the latter situation, he inoculated a boy, named Phipps,

It may be interesting hereafter, as a chronological fact, to know that this important experiment took place on the 14th of May, 1796; and such have been the results of what was done on that day, in an obscure country hamlet, that at this moment the discovery is gone abroad over the whole inhabited world, and the name of Jenner, or, (in strange tongues), a sound imitating his name, is now articulated through the world by every "kindred, and tongue, and nation,"

"Familiar in their mouths as household words."

We gladly pass over the two succeeding chapters which give an account of the first promulgation of Dr. Jenner's discovery, and the obloquy, the disingenuousness, the calumny, and the controversy, with which it was received, as well by its early proselytes, as its staunch opposers. The author gives a very animated *catalogue raisonnè* of the various tempestuous pamphlets which were showered from the press by *Mosely, Birch, Rowley*, and others, in the insanity of their vain opposition; and refutes with much clearness, the arguments, if arguments they can be called which arguments were not, they employed to throw terror and prejudice into the minds of the lower classes against vaccination. We shall transcribe his concluding paragraph, which may be regarded as the summing up of his lengthened reasonings against these furious alarmists.

"Whether the lymph was taken from a cow, or from the human subject, the malady produced is simply the vaccine; and respectable observers have never detected any other effect from vaccination. There are not even those grounds of suspicion which are attached to variolous inoculation; for the vaccine process is so gentle, as neither to enfeeble the habit, nor to rouse into action any indisposition which may be lurking in the constitution; and its influence is so transient, that in a very few days even delicate infants recover their pristine health." p. 66.

The 4th chapter treats of small-pox occurring after vaccination; and of small-pox and several other infectious diseases, in some instances, recurring to the same individuals. This is a most valuable portion of the work; and besides abounding in deep, independent, and original ideas about disease in general, it will, we think, prove highly interesting to an intelligent reader, from the train of useful reflection in his own mind, which Mr. Moore's casual and aphoristic hints about obscure points of physiology, pathology and therapeia, must infallibly awake and set in motion. We extract the followiug specimen of his reasoning, regretting that our limits will not permit us to do it ampler justice.

"It has been observed, that there are certain maladies which are apt to afflict the same persons repeatedly; and the oftener they have taken place, the more prone they are to recur. Goût, rheumatism, catarrh, and ophthalmia, are of this kind. Yet, notwithstanding the general truth of this position, there are some persons who have had one attack of these maladies, and through a long life have never had a second.

"To contrast with this, there is another class of diseases which mankind in general are susceptible of contracting only once. But each of these diseases have (has) assailed some individuals more than once; except one, whose first attack is supposed to be always mortal.

"Diseases of this second class are all produced by morbid poisons, either in a liquid or gaseous form; and are certainly of a less ancient origin than those distempers which are excited by cold, heat, moisture, surfeits, want, marshy vapours, and other causes, whose existence has been co-eval with the world.

"The most terrible of the morbid poisons is the saliva of a dog affected with hydrophobia. Whenever this deleterious fluid has been deposited in a wound, and has begun to stimulate the living fibre, the human powers are quite inadequate to expel, or to resist it: and as no remedy has been found out capable of controlling its violence, the disease continues until the vital powers are extinguished.

"The syphilitic poison is also superior to the medical powers of Nature, and would be as fatal as the hydrophobia, if an antidote had not fortunately been discovered to counteract its virulence. But as this disease can only be cured by the operation of medicine, and not by the natural actions of the body, as soon as the influence of the medicine has ceased, the body again becomes susceptible of the disease; and this malady may be contracted again and again by repeated applications of the contagion.

"The other morbid poisons, the plague, small-pox, chicken-pox, measles, hooping-cough, mumps, the scarlet, and perhaps some other fevers, are all distinct infections, yet regulated by similar laws. When a man in health contracts, for the first time, any of those infections, he is seized at a regular period with the peculiar symptoms of the malady; and there is formed in the contaminated body abundance of infectious vapour, which evaporates from his person. The disease sometimes proceeds in a tranquil, and at other times in a most tumultuous course. But if its progress is not interrupted by death, after it has reached to a certain height, and when infection is steaming from every pore, the symptoms meliorate, the production of the morbid matter declines and stops; that which was formed is eliminated; the body becomes insensible to the poison, and gradually resumes a state of health.

"Unless this alteration in the body took place, all these maladies would be mortal in every instance; for there is no specific for any of them known.

"The old physicians, either from reluctance to acknowledge their incapacity of accounting for these favourable events; or from a facility of admitting mysterious words as adequate causes, imputed the whole to the medical powers of Nature. This continues, to the present times, a favourite phrase, and is even sometimes styled a Doctrine. But whatever produces that insensibility to the stimulus of these poisons, which is acquired in the progress of these diseases, the altered state of body usually continues through the remainder of life. Individuals, therefore, who survive one attack of these distempers, commonly resist the infection ever afterwards. This general maxim, however, like all others, has its exceptions. It is universally admitted, that the plague has frequently attacked the same persons repeatedly; and that the hooping-cough, mumps, and scarlet fever have sometimes seized the same individuals oftener than once, is rarely denied. But the recurrence of the measles has been disputed. This scepticism, however, is not certainly to be found in the very early writers, most of whom admit the occasional exception; and the evidence of later times is quite decisive. Richard Morton saw one case where the measles occurred twice; Professor De Haen *(Ratio Medendi de Haen)* attended two patients of the same kind; and Burserius *(Instit. Med. Pract. Burser. tom. 2)* has collected a number of examples, from unquestionable authority, where the measles took place twice. In addition to which, Dr. Baillie, a physician of the most clear and unbiassed judgment, lately observed, and distinctly described* eight examples of this incident; since which, this question seems to be considered as settled.

"That the small-pox was also governed by the same general rules was never doubted, until variolous inoculation and vaccination became subjects of medical feuds. But the friends of inoculation in the middle of the last century, and the enemies of vaccination of the present day, have ventured to deny that small-pox ever had attacked the same persons twice; which denials are in opposition to recorded affirmations from the highest authorities. The profession, indeed, of late, has been excited to a close consideration of these points; and a multitude of examples has been published of small-pox having seized certain persons twice; which facts are so common, as no longer to excite particular attention." P. 71—74.

Our author is also of opinion, that though the effects of the vaccine virus upon the living body are far less virulent, still that they are regulated by the same general principles as the small pox and all other morbid poisons.

---

* Transactions of a Society for the Improvement of Medical and Chirurgical Knowledge, vol. iii.

In fact, he argues, and with much probability, for such a radical resemblance between the two, that the vaccine lymph and variolous pus maintain a reciprocal control over the actions of each other; hence he concludes, that the former will never fail to prevent the small-pox, except in those very rare and peculiar habits which are susceptible of contracting the small-pox oftener than once.

Now, though his reasoning is ingenious, we fear that his premises are faulty, or that he has over-looked something in this difficult discussion; for it does appear to us, either that his conclusion is rather premature, or else that those peculiar habits, susceptible of second attacks of small-pox, are not so rare as he represents them. We admit, that the alleged are far more numerous than the actual failures of vaccination; that ambiguous eruptions, or severe cases of chicken-pox, occurring after cow-pox inoculation, are eagerly caught at, and sounded abroad, by the ignorant and the interested, as proofs of the inadequacy of the preventive: we can allow, also, full force to the fact he states, that out of 2,671,662 persons in France, who had been properly vaccinated, only seven had afterwards taken the small-pox; nay, we can add another strong fact, of which we have been credibly informed, viz. that in *Amsterdam*, out of a population of 200,000 souls, such has been the generality of the success of vaccination, that, during the first part of the past year, not an individual case of variola has appeared:—but what shall be said to Dr. Dewar, who brings forward 54 cases of small-pox (visited by him in the town and neighbourhood of Cupar) occurring after the subjects had gone through the vaccine disease? Only ten of these, it is true, were inoculated by medical practitioners; but Dr. D. found no particular reason for disputing the genuineness of the vaccine disease which the other 44 patients had undergone. It appeared to him, that the disease, in this particular epidemic, attacked indiscriminately the vaccinated and unvaccinated; and although, in the former, it was generally milder, and the secondary fever often absent, still it was not always so; for some of them, those too who had been vaccinated under the superintendence of medical men, had the disease in a form of great severity. From all he saw, Dr. Dewar is of opinion, that

"It is evident vaccination did not ultimately prove a certain preventive of an attack of small-pox; yet it evidently gave, in some instances, a remarkable temporary or occasional protection. On this point we have still much to learn; and, in the mean time, must remain content with the general conclusion, that vaccination gives a probable, but not a certain, protection from an attack of small-pox; and in this respect it must even be admitted, that we find a marked distinction between it and the variolous inoculation." P. 32—33.

These things we recommend to the consideration of Mr. Moore: for our own parts, satisfied as we are of the general preventive power of vaccination, we leave the occult causes which produce occasional failures to be developed by time; for in the present state of our knowledge we have as yet too few facts.

The author's fifth chapter is on the subject of varicella or chicken-pox; and he evinces very considerable learning and research in tracing the notices and descriptions of it that are to be found in the early writers, both Arabian and European. He also enters at great length into the diagnosis between this disease and the genuine small-pox; but it is a difficult subject, and it is scarcely any discredit to him when we say he has failed of being clear. Verbal descriptions, though ever so accurate, can seldom teach what can only be rightly known by actual and careful inspection. No doubt, when each of these two diseases appears in its proper colours, the distinction is sufficiently apparent; but when small-pox is peculiarly mild, and chicken-pox extraordinarily violent, which is sometimes the case, then all the discriminating marks are obscured. The perplexity is increased, too, by our knowledge of the fact, that a new kind of small-pox, different from the mild species, has lately been observed in persons who had previously undergone vaccination; and that this malady neither proceeds according to the regular course of small-pox nor chicken-pox, but appears like a new and intermediate eruption.

We regret that our limits compel us entirely to pass over the chapters describing the reception of vaccination with the public in England—the parliamentary proceedings with regard to the remuneration bestowed on Dr. Jenner—the gradual establishment of vaccine institutions—the extension of the blisful discovery, and its consequent benefits through Scotland, Ireland, and all the British dominions and dependencies in the East and in the West—and its ultimate diffusion through Germany, Prussia, Russia, Sweden, Denmark, France, Switzerland, Italy, Spain, Persia, Africa, and America. This part of the work contains much curious and amusing matter; but, as it is less immediately connected with medical science, we must pass at once to the concluding chapter of this valuable volume, which has for its subject the right mode of performing vaccination.

Owing to the irritability of new-born infants, and the uncertainty of their organization being perfect, our author thinks the operation of inoculating with the vaccine lymph should be deferred till three weeks after birth; but should small-pox contagion be raging in the neighbourhood, the operation should be performed earlier, *coute qui coute*, for the danger of it is little, compared with the danger of infection.

The only thing material with regard to this operation is, to ascertain that the vesicle has not acted locally, but effected the desired change on the constitution; this is a matter of great importance, and has attracted to it, from practitioners, an adequate degree of attention. Hence has originated the practice of testing, first set on foot by Mr. Pearson of London, and Mr. Bryce of Edinburgh, who simultaneously, but without any knowledge of what was doing by each other, proposed to try the effect of re-vaccination, during every period of the progress of the vaccine vesicle.

We are inclined to think that this practice was suggested by what was usually done, in the last age, by practitioners who inoculated for the small-pox. They were wont to make a puncture every day, until the constitutional affection appeared, and were always much surprised to find, that, when this wished-for affection *did* appear, all the successive punctures became ripe at the same time. Mr. Bryce, therefore, punctured the first vesicle on the 5th or 7th day, took lymph from it, and made a second puncture on a part of the body different from the former. If the first had taken proper effect, the second resembled it in miniature, was accelerated by it, got ripe at the same time, and both desiccated together. If it had not, the second healed without any such effects.

Mr. Dunning, of Plymouth, first pointed out the danger of puncturing and draining a vesicle of its lymph, as he conceived such practice left the patient, if originally of a high variolous susceptibility, still liable to be infected with the small-pox; and our author coincides with him in opinion, as the following quotation will better show:

"In vaccination, the governing principle, which ought always to influence the surgeon, is to infect his patient most thoroughly with the virus. Little is to be feared from any excess of the vaccine fever; but an imperfect constitutional infection may produce a false security, and may diminish or frustrate the benefit expected from the operation. When three or more vesicles have been excited, lymph may be taken from this subject. But it is prudent always to leave two complete vesicles to pass through their course untouched." P. 290—1.

Mr. Moore seems, therefore, to lay very little stress on Mr. Bryce's test; while Dr. Dewar, on the other hand, thinks it ought to be invariably practised:

" Who shall decide," &c.

For our parts, we cordially agree with the latter gentleman, because we think the *test* rests on a philosophical principle, and has, moreover, been sealed by experience. All objections will be removed, if three punctures are made originally on different parts of the body, and the lymph for testing be taken from this third vesicle, leaving the others to go through their course unpunctured and unmolested.

The manner of performing this little operation is so well known to every body, that we extract the following quotation (and it shall be the last), not for the sake of the caveat it conveys, but merely as a specimen of the serio-ludicrous tone which so frequently appears throughout the volume.

" The prudent will not only avoid the perils proceeding from negligence and cold indifference, but will also eschew the practice of a most zealous clergyman of the Methodist persuasion, whom I once saw operate.

" This worthy man grasped his lancet firmly, but not after the fashion of surgeons. He continued alternately taking lymph from one infant, inserting it into another, and expounding his doctrine. A moment's pause occurring in his discourse, I seized the opportunity, and, to stop a work of super-vaccination, asked, ' how many punctures he deemed necessary ?' He proceeded with fluency, ' So innocent is the lymph, so transitory its workings, and so lasting its effects, that, be assured, you cannot pour too much into the flesh.'—In pronouncing these words, he impressed the epithets on his hearers with an elevation of the voice, and on the child with a depression of the lancet, who shrieked at each gesticulation. Yet the mother, who would have been infuriate had a surgeon extorted such screams, looked quite placidly at her revered pastor; being inwardly convinced, that all the pains taken and given by him, would, in some mysterious way, do good to her suckling.—As surgeons cannot expect to meet with the same same indulgence, they are recommended to be more merciful in their mode of operating." P. 292.

Here we must close our analysis of these two treatises. Of Mr. Moore's we have sufficiently spoken;—yet few readers of taste will be content, we presume, with our meagre extracts, when the original work itself is to be had. It must be eminently serviceable to the cause of vaccination in times like these, when prejudice and misrepresentation are abroad. Dr. Dewar's little tract, also, will not be without its weight, inasmuch as it is written with the view of preventing the forging of some rumours, and the gross exaggeration of some facts and occurrences which were likely to arise from what he had witnessed; and of holding forth "the favourable features of vaccination which remain unaffected." He is a gentleman of great talents, united with great industry, and, from his addiction to the "musæ severiores," is well qualified for any intricate philosophical investigation, where a union of acuteness and cautiousness are requisite.

May we be permitted to hope, that truth will at last triumph; that vaccination will at last prevail; and that future generations will only be traditionally acquainted with a disease which was wont to sweep off one tenth of the human race—a disease to which the touching language of a modern classic might, a few years back, have been well applied: "Pauci sunt quibus cognati, familiares, aut amici, hac febre abrepti, non sunt lugendi. Misera hæc pestis, sæva, atrox, et insensibilis, teneros et amabiles depascens, cæde et luctu patriam implet."*

* Gregory, De Morbis Cœli mutatione medendis, p. 42.

